# Biology of endophilin and it’s role in disease

**DOI:** 10.3389/fimmu.2023.1297506

**Published:** 2023-12-05

**Authors:** Lu-Qi Yang, An-Fang Huang, Wang-Dong Xu

**Affiliations:** ^1^ Department of Evidence-Based Medicine, Southwest Medical University, Luzhou, Sichuan, China; ^2^ Department of Rheumatology and Immunology, Affiliated Hospital, Southwest Medical University, Luzhou, Sichuan, China

**Keywords:** endophilin, diseases, function, endocytosis, autophagy

## Abstract

Endophilin is an evolutionarily conserved family of protein that involves in a range of intracellular membrane dynamics. This family consists of five isoforms, which are distributed in various tissues. Recent studies have shown that Endophilin regulates diseases pathogenesis, including neurodegenerative diseases, tumors, cardiovascular diseases, and autoimmune diseases. *In vivo*, it regulates different biological functions such as vesicle endocytosis, mitochondrial morphological changes, apoptosis and autophagosome formation. Functional studies confirmed the role of Endophilin in development and progression of these diseases. In this study, we have comprehensively discussed the complex function of Endophilin and how the family contributes to diseases development. It is hoped that this study will provide new ideas for targeting Endophilin in diseases.

## Introduction

The Endophilin family is a group of protein containing SH3 (Src homology 3) structural domain and BAR (Bin-amphiphysin-Rvs) structural domain. This family was first discovered in 1996 (1). It consists of two subfamilies, including Endophilin A and Endophilin B. Endophilin A consists of three isoforms: Endophilin A1, Endophilin A2 and Endophilin A3. Endophilin B consists of two isoforms: Endophilin B1 and Endophilin B2. Endophilin A regulates endocytosis through its C-terminal SH3 domain and N-terminal BAR domain. Endophilin B1 (Bax interacting factor-1, Bif-1) interacts with Bax to promote release of cytochrome C in mitochondria (2). Endophilin B1 also activates the apoptosis-related signaling pathway to promote cell apoptosis (2). Endophilin B1 induces autophagy by forming a complex with Beclin-1, and then activates phosphatidylinositol 3-kinase (PI3KC3) signaling (3). Expression of Endophilin in different diseases was different, for instance, Endophilin A1 expression was increased in brain of patients with Alzheimer’s disease and Parkinson’s patients (4, 5). Endophilin A1 expression is down-regulated in colon cancer, uroepithelial carcinoma. Endophilin A2 may be protective against cardiovascular diseases. Endophilin A3 was associated with a poor prognosis of patients with colorectal cancer, where there was higher expression of Endophilin A3 in patients with more advanced disease (6). Endophilin B1 may inhibit disease development through induction of autophagy and apoptosis. In recent years, Endophilin has attracted widespread attention as an important endocytosis protein. In this study, we reviewed the biological functions of Endophilin family and discussed their roles in diseases.

## Discovery

After screening cDNA library of mouse embryonic cells, a total of 18 different SH3 structural domain-containing proteins were identified. Among the proteins, 10 of which had not been previously reported, such as SH3P4, SH3P8, SH3P13 (1). On the other hand, the gene encoding for human Endophilin was originally cloned from an acute myeloid leukemia case at chromosome 9p13. The case was named EEN (Extra Eleven Nineteen) due to the fact that it had a translocation of chromosomes 11 and 19, which allowed the gene to form a fusion gene with mixed lymphocytic lymphoma (MLL) (7). There were two homologous sequences named EEN-B1 and EEN-B2, respectively. EEN-B1 was homologous to the mouse SH3P4 gene, and EEN-B2 was homologous to the mouse SH3P13 gene. Finally, the protein was named Endophilin either in animal or human based on the affinity for several endocytosed proteins (8, 9). Moreover, a novel cDNA was identified by two-hybrid screening using yeast clones, and the gene was named SH3GLB1 (10). SH3GLB1 interacted with SH3GLB2, which had 65% amino acid homology with SH3GLB1 (11). SH3GLB was renamed Endophilin B1 and Endophilin B2 due to its high similarity to the Endophilin family, especially the SH3 structural domain.

## Subtypes and structure

### Subtypes and distribution

Endophilin A1 (SH3P4 or SH3GL2) is dominantly expressed in brain tissue (12). Endophilin A2 (SH3P8 or SH3GL1 or EEN) is distributed in different tissues and organs, such as pancreas, placenta, prostate, testicles and uterus. Endophilin A3 (SH3P13 or SH3GL3) is expressed in brain and testicular tissues (13, 14). Subfamilies of Endophilin A are mainly localized in cytoplasm of cells. Endophilin B1 is highly expressed in heart, skeletal muscle, kidney, and placenta (15). Endophilin B1 is mainly localized in intracellular membrane, and Endophilin B1 is also localized at mitochondrial membranes, golgi membranes and autophagosomal membranes (16). Endophilin B2 is expressed in skeletal muscle, adipose tissue, lung, brain and mammary gland (17).

### Structure

SH3 structural domain is connected to the BAR via a variable length splice region (18). The SH3 structural domain recognizes and binds proline-rich structural domain, including synaptic proteins and dynamin proteins (15, 19, 20). N-BAR structural domain is required for binding lipid bilayer and inducing membrane bending (21, 22), and can induce and stabilize membrane curvature upon dimerization (23–25). N-BAR has three major functional regions, including N-terminal amphiphilic helix (H0), amphiphilic helix inserted into helix 1 (H1), and crescent-shaped body formed by the dimerized BAR structural domain. H0 mediates membrane binding, H1 inserts into the membrane and cooperates with the dimerized BAR body to drive membrane curvature (26–29).

## Biological function

### Endophilin A

**Figure 1:**
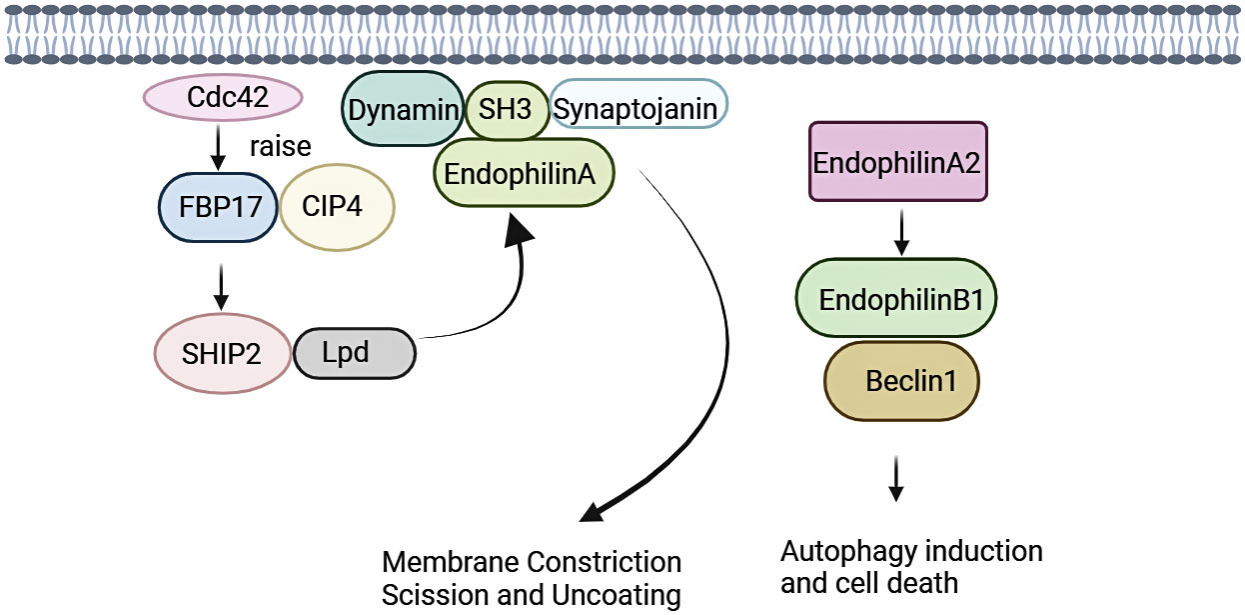
Signalings of Endophilin A. Endophilin A binds to synaptic proteins and dynamin proteins through the SH3 structural domain, and is necessary for membrane rupture and vesicle release. Endophilin A2 overexpression promotes interaction of Bif-1 with Beclin-1. This process will promote formation of autophagosomes, thereby promoting autophagy.

Endocytosis regulates function of eukaryotic cells, including physiological processes such as nutrient uptake, signal transduction, and cell growth. Family of Endophilin induces and stabilizes membrane curvature in the endocytic pathway (13). Endophilin A is involved in Clathrin-mediated endocytosis pathway (CME), which regulates synaptic vesicle formation, including Clathrin-encapsulated vesicle outgrowth, division, and decapsulation (12, 30). SH3 structural domain and N-BAR structural domain of Endophilin A performs significantly in this process. Endophilin A is necessary for membrane rupture and vesicle release, where SH3 structural domain binds to proline-rich structural domain of other endocytosed proteins, including synaptic proteins and dynamin proteins. SH3 structural domain assembles around the necks of Clathrin-encapsulated pits with initiators (31–33). Recent studies have shown that the N-BAR structural domain of Endophilin A possesses lysophosphatidic acid acyltransferase (LPAAT) activity. The lipid membrane curvature required for vesicle formation was induced under this activity.^14^ Endophilin A1 and Endophilin A2 positively regulate endocytosis, whereas Endophilin A3 may negatively regulate Clathrin-mediated endocytosis. Endophilin A3 inhibits Clathrin-mediated endocytosis of transferrin, which is overexpressed in cos-7 cells. Endophilin A3 co-localizes with dopamine D2 receptors in olfactory nerve terminals, inhibits dopamine D2 receptor-mediated endocytosis, and promotes cells differentiation (**Figure 1**) (34).

Endophilin A is required for fast Endophilin-mediated endocytosis (FEME) (35). FEME is triggered by a series of molecular events, and this event is initiated by Cdc42 (36). Cdc42 recruits Cdc42-interacting protein4 (CIP4)/formin binding protein17 (FBP17), which engages SH2-containing inositol phosphatase 2 (SHP2) and lamellipodin (Lpd). Endophilin A binds to the Lpd proline-rich region, concentrating Endophilin into clusters at discrete sites in the plasma membrane. Liquid-liquid phase separation (LLPS) is a key mechanism for protein assembly during FEME. LLPS promotes the formation of dynamically enriched clusters on the membrane that can act as initiation sites for FEME. The liquid-like clusters then recruit additional endocytose proteins such as activated receptors. The LLPS in Endophilin is mainly driven by the BAR domain, and the SH3 domain also promotes the binding of proline-rich-motifs (PRMs) proteins to the droplet (37, 38). In the absence of receptor activation, the clusters disassemble rapidly following local recruitment of the Cdc42 GTPase-activating proteins RICH1, SH3BP1, or Oligophrenin-6. Then, a new cycle was formulated, and was continuously prepared for FEME. After receptor activation, the ligand recognizes the receptor. Endophilin can bind directly to the receptor or indirectly via adaptin, which then form the FEME carrier. A number of membrane protein receptors were rapidly internalized by FEME signaling, including G-protein coupled receptors [for instance, β1-adrenergic receptor, (β1AR)], receptor tyrosine kinases [for instance, epidermal growth factor receptor, (EGFR)], cytokine receptors (39, 40).

Endophilin A2 may function as a potential activator of autophagy. Endophilin A2 overexpression promoted interaction of Bif-1 with Beclin-1. Then, formation of autophagosomes was induced, which would promote autophagy (41).

### Endophilin B

**Figure 2:**
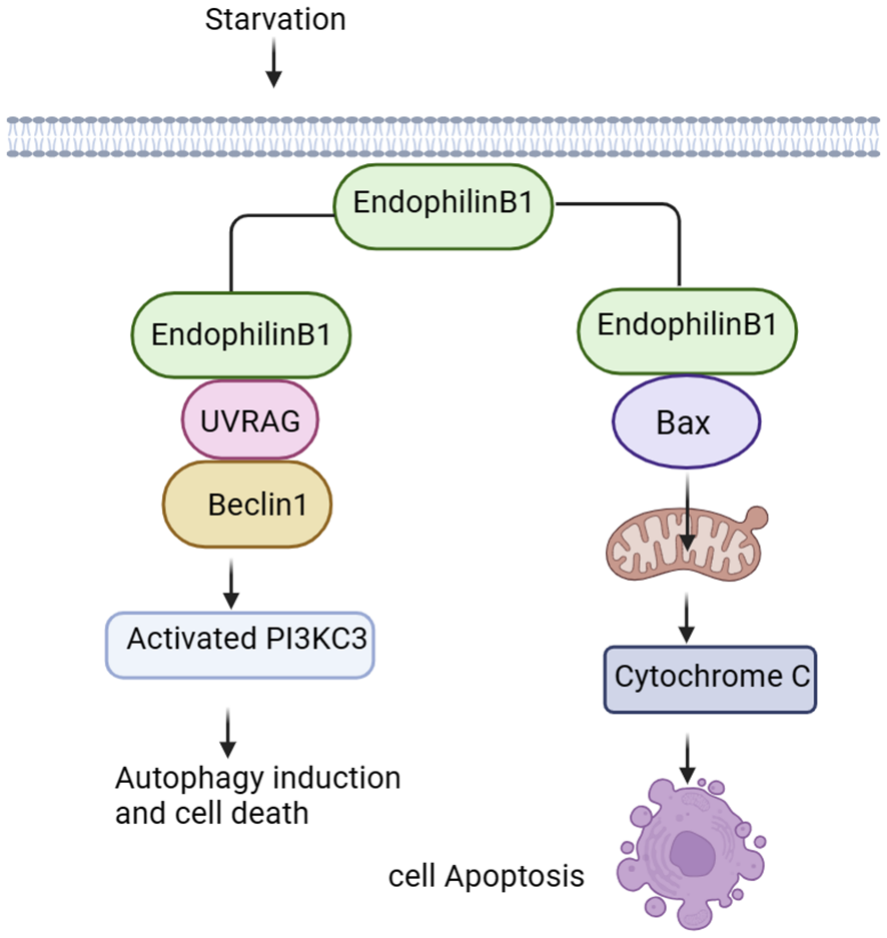
Signalings of Endophilin B. Endophilin B1 forms a complex with Beclin-1 through UVRAG and activates PI3KC3, thus inducing autophagy. Bif-1 interacts with Bax to promote release of cytochrome C in the mitochondria, activating the apoptosis-related signaling pathways.

Endophilin B1 is a multifunctional protein involved in apoptosis, mitochondrial morphological changes and autophagosome formation (**Figure 2**). Bif-1 was initially identified as a pro-apoptotic protein and acted as a novel Bax activator to control apoptosis in the mitochondria (42). In response to apoptotic stimuli, Bif-1 translocates from cytoplasmic lysate to mitochondria, inserts into the outer mitochondrial membrane (OMM), and forms oligomeric complexes. Complexes of Bif-1 alter morphology of monolayer vesicles of the cell membrane, leading to formation of liposome vesicles and altering membrane curvature (43). Interaction between Bif-1 and Bax changes in a time-dependent manner. Bax also translocates to mitochondria to promote conformational changes, leading to alteration in the permeability of the OMM and release of apoptosis-related factors, such as cytochrome C. This process will activate apoptosis-related signaling pathways and promote cells death (44, 45). It was found that Bax could not oligomerize Endophilin B1, which lacks C-terminal SH3, suggesting that the SH3 structural domain at the C-terminus is important for interaction of Bif-1 and Bax as well as for the oligomerization of Bif-1 (2).

Autophagy is an evolutionarily conserved cellular process that initiates extensive degradation of cytoplasmic components in response to environmental changes (2). The degradation process involves in dynamic membrane rearrangements, leading to formation of double membrane autophagosomes. During starvation, autophagosomes provide nutrients to eliminate proteins and organelle through lysosomal degradation, maintaining intracellular homeostasis (46, 47). Bif-1 binds to polyproline structural domain (PR) of the ultraviolet radiation resistance-associated gene (UVRAG) via the SH3 structural domain, and then bridges Beclin-1. Finally, Bif-1 forms a complex with Beclin-1 to bend the N-BAR structural domain, resulting in activation of PI3KC3 and induction of autophagy (48, 49). Vesicle nucleation is an early step in autophagosome formation, and Bif-1 has intrinsic membrane-bending induced activity (50), suggesting that this protein may play a role in biogenesis of autophagosomal membranes. Interestingly, Bif-1 aggregated in cytoplasmic lysate puncta and co-localized with Atg9 during induction of autophagy, implying that Bif-1 together with the UVRAG-Beclin-1-PI3KC3 complex could regulate Golgi complex Atg9 vesicles to form autophagosomal membranes (14, 51). Formation and transportation of these vesicles is essential for the biogenesis and expansion of the autophagosome membrane during induction of autophagy. Bif-1 co-localizes with Atg5 and LC3. Atg5 is expressed on phagosomes in the early stage of autophagosome formation, and LC3 is a characteristic marker of autophagy (49). These results suggest that Bif-1 is involved in the early stage of autophagosome formation and may play a role in autophagosome biogenesis or amplification (**Figure 2**).

## Diseases

### Neurodegenerative diseases

#### Alzheimer’s disease

Alzheimer’s disease (AD) is a neurodegenerative disease (4). Its pathological features include Aβ deposition-induced senile plaques, excessive accumulation of Tau proteins and neurofibrillary tangles, neuronal and vascular deformities, endothelial cells (EC) dysfunction, and disruption of the blood-brain barrier (BBB) (52). Studies have shown that Aβ is the causative factor of AD and contributes to cognitive deficits in AD. Endophilin A1 expression was significantly increased in brain of AD patients compared with that in healthy controls (**Table 1**) (53). Endophilin A1 expression was significantly increased in the brain of AD patients compared to healthy controls. Increased Endophilin A1 expression activates the JNK signaling pathway, which subsequently induces Aβ expression, leading to mitochondrial dysfunction and neuronal death, ultimately leading to AD. Increased JNK activity phosphorylates Tau proteins, leading to neuroinflammation (4). Furthermore, increased Endophilin A1 promotes expression of reactive oxygen species (ROS), activates p38 signaling, which then contribute to Aβ-induced mitochondrial dysfunction and synaptic damage (69). Extracapsular shedding of amyloid precursor protein (APP) is a key process in Aβ production. Endophilin A3 decreases rate of APP endocytosis and accelerates Aβ degradation by promoting APP shedding (64). Loss of Endophilin B1 may exacerbate pathology of AD, where there was decreased expression of Endophilin B1 in AD patients’ brain tissues, brain tissues from AD mice, and Aβ-treated neurons. Deficiency of Endophilin B1 in AD mice exacerbated amyloid-induced plaquelike, Tau protein phosphorylation, astrocyte hyperplasia, cognitive decline and synaptic deformation. Overexpression of Endophilin B1 in mice cortical neurons prevents Aβ-induced mitochondrial dysfunction (70). In the mice with middle cerebral artery occlusion/reperfusion (MCAO/R) model, Bif-1 gene deficiency led to larger infarcted areas. Neurons from Bif-1 gene knockout mice contained fragmented mitochondria. Similarly, knockdown Bif-1 gene in wild-type neurons also showed fragmented mitochondria, which were more depolarized, indicating mitochondrial dysfunction. These data suggest that Bif-1 is essential for maintaining mitochondrial morphology and function in neurons compared with non-neuronal cells, and Bif-1 deficiency renders neurons more susceptible to apoptotic stress (71, 72). Therefore, Endophilin A1, A3 and B1 may be new therapeutic targets for AD.

**Table 1:** Expression profile of Endophilin in different diseases.

Cytokine	Year	Author	Disease	Sample	Expression	Reference
Endophilin A1	2008	Ren et al.	Alzheimer's disease	Brain tissue^a^	Increased	(53)
	2019	Connor-Robson et al.	Parkinson's disease	Brain tissue^a,b^	Reduced	(54)
	2013	Majumdar S et al.	Urothelial cancer	Bladder tissue^a^	Reduced	(55)
	2008	Sinha et al.	Breast cancer	Breast tissue^a^	Reduced	(56)
Endophilin A2	2007	Ma LH et al.	Leukaemia	Serum^a^	Increased	(57)
	2016	Guan H et al.	colorectal cancer	colorectal tissue^a^	Increased	(58)
	2012	Matsutani T et al.	glioblastoma	Serum^a^	Increased	(59)
	2017	Baldassarre T et al.	breast cancer	breast tissue^a^	Increased	(60)
	2017	Li EQ et al.	osteosarcoma	osteosarcoma tissues^a,b^	Increased	(61)
	2016	Huang EW et al.	Atherosclerosis	Plasma^a^	Increased	(62)
	2021	Norin et al.	Rheumatoid arthritis	Whole blood^a^	Increased	(63)
Endophilin A3	2016	Liu W et al.	Parkinson's disease	Endothelial cell^a^	–	(64)
	2018	Poudel et al.	Colon cancer	Colon tissue^a^	Increased	(6)
Endophilin B1	2011	Wong AS et al.	Parkinson's disease	Brain tissue^b^	Reduced	(65)
	2022	Mohammadi et al.	Breast cancer	Breast tissue^a^	Reduced	(66)
	2008	Coppola D et al.	Prostate cancer	Prostate tissue^a^	Reduced	(67)
	2016	Xu L et al.	Rectal cancer	Prostatic tissue^a^	Reduced	(45)
	2020	Frangež et al.	Melanoma	Melanoma biopsy^a^	Reduced	(68)

a Human.

b Mice models.

#### Parkinson’s disease

Parkinson’s disease (PD) is the most common neurodegenerative movement disorder. PD is characterized by loss of dopaminergic (DA) neurons in the substantia nigra and accumulation of α-synuclein (73). Endophilin A1 expression is related to leucine-rich repeat kinase 2 (LRRK2), α-synuclein expression in PD patients (74). Endophilin A1 gene knockout mice showed endocytosis defects at the synapse, eurodegeneration, and up-regulation of Parkin, an E3 ubiquitin ligase related to PD (75). Knockdown or pharmacological inhibition of LRRK2 results in defective synaptic vesicle endocytosis (SVE), altered synaptic morphology and impaired neurotransmission (76). Interestingly, it was found that the levels of Clathrin and Endophilin A1 protein were significantly reduced in brain of PD patients with LRRK mutation. LRRK2 phosphorylates Endophilin A1, altering its membrane structure and causing dysregulation of the endocytosis pathway (54). *In vitro* and *in vivo* experiments showed that LRRK2 phosphorylates Endophilin A1 at serine 75 (S75) and at threonine 73 (T73), which regulates membrane remodeling and endocytosis activity of Endophilin A1, disrupts SVE and neurotransmission, and ultimately leads to PD development (46, 77). A recent study found that expression of Endophilin A1 in lipopolysaccharide (LPS)-induced substantia nigra from PD mice was increased, which promoted occurrence and development of PD. Endophilin A1 gene silencing reduces the production of ROS, inhibits the activation of NOD-like receptor protein 1 (NLRP1) inflammasomes, reduces the release of inflammatory factors and damages of dopaminergic neurons (78). Genome-wide association studies have shown that the rs13294100 polymorphism in the Endophilin A1 gene is associated with PD susceptibility and may be a risk locus for PD in European ancestry (79). These results suggest that Endophilin A1 may relate to development of PD. Accumulation of autophagy is a hallmark of neurodegenerative diseases (80). Autophagy causes neurodegeneration (81). Autophagy dysregulation is associated with PD, and accumulation of autophagosomes was observed both in PD patients’ brain and in PD mice models (82). Autophagy is critical for clearance of α-synuclein aggregates, but excessive autophagy is also associated with neuronal loss (83). Starvation and overexpression of α-synuclein increases the level of Cdk5/p35-induced phosphorylation of Endophilin B1 Thr145 in neurons. Phosphorylation of Endophilin B1 promotes its dimerization and recruitment of the UVRAG/Beclin-1 complex to induce autophagy, leading to neuronal death (65). In response to calcium influx, Endophilin A1 acquires structural flexibility and diffuses to the synaptic cavity and drives autophagosomes formation. Genetic mutation in SH3GL2 gene blocks autophagy by disrupting the calcium sensitivity of SH3GL2. The process will form a fixed protein that does not respond to calcium influx, thereby disrupting autophagy induction at synapses (84, 85).

Endophilin A1, A2, and A3 gene knockout mice showed impaired synaptic transmission, leading to perinatal death. Endophilin A1 knockout mice have a normal phenotype, suggesting functional compensation for Endophilin A2 and Endophilin A3. In contrast, Endophilin family gene partially knockout may result in severe neurological defects, including epilepsy and neurodegeneration (86).

#### Tumors

In recent years, more studies focused on role of Endophilin in cancer development, along with expectation for finding out promising new targets for cancer and useful prognostic indicators for cancer.

Endophilin A1 is a tumor suppressor and its expression was down-regulated in many cancers, including colorectal, uroepithelial, and breast cancers (6, 55, 56). Silencing expression of Endophilin A1 in cell line RT4 promoted cells proliferation, colony formation, suppressed EGF-induced EGFR internalization (55, 87). Injection of Endophilin A1 gene knockout cell line RT4 into urothelial carcinoma mice promoted impairment of oncogenic behaviors (88). Studies have shown that Endiphilin A3 expression is associated with poor prognosis in colon cancer patients, and more advanced colon cancer patients had higher expression of Endophilin A3. Endophilin A3 promotes tumor cells (U2OS cell line) proliferation through enhanced endocytosis, as well as stimulates U2OS cell line migration through activation of Rac1 small GTPase (6). Endophilin A3 activates endocytosis pathway, causing U2OS cell line to down-regulate surface expression of CD166, improve migration and adhesion properties (89). This pattern of endocytosis will promote hyperproliferation or metastasis of U2OS cell.

Endophilin A2 is the only one member of Endophilin family expressed in hematopoietic cells (57), and acts as a fusion partner of the MLL gene. EBP is a novel EEN-binding protein, which is mainly expressed in the cytoplasm. EBP interacts with the SH3 structural domain of EEN through proline-rich motif PPERP, then forms a stable trimeric complex by recruiting SOS2. The formed complex will inhibit Ras signaling-induced cellular transformation and Ras-mediated activation of ELK-1 transcription (90–92). Endophilin A2 is highly expressed in tumors including colorectal cancer, glioblastoma, breast cancer, and osteosarcoma (58–61). Inhibition of Endophilin A2 enhances chemosensitivity of colorectal cancer cells through downregulation of multidrug resistance protein (MDR1), by which the effects were mediated via the epidermal growth factor receptor (EGFR)/extracellular signal-regulated kinase (ERK)/activator protein (AP-1) pathway (58). In high-grade glioblastoma, aberrant expression of Endophilin A2 induces a systemic immune response. Endophilin A2 linked EGFR to activate the RAS pathway, leading to activation of the MAPK cascades. This further leads to altered expression of genes associated with cell proliferation (59). Reduced expression of Endophilin A2 could inhibit activation of the Akt/GSK-3b/FAK signaling pathway, leading to reduced cycling-D1 expression, weakened activation of P-Rb, and cell cycle arrest in the G0/G1 phases, which in turn regulated cell proliferation (61). Thus, EndophilinA2 may pass through multiple signaling pathways and thus participate in tumor development.

Endophilin B1 gene deficient mice showed spontaneous tumor development, and knockdown of Endophilin B1 promoted growth of Hela cells, suggesting that Endophilin B1 may suppress tumorigenesis. Interestingly, there was abnormal expression of Endophilin B1 in cancer-related tissues compared to adjacent healthy tissues, including breast cancer, prostate cancer, rectal cancer and melanoma (44, 45, 66–68). Deletion of Bif-1 may inhibit apoptosis and promote tumorigenesis. Endophilin B1 accelerates Bax degradation by binding to Bax and enhances apoptosis-induced kinetics in response to innate apoptosis-related signaling, which will increase the permeability of the outer mitochondrial membrane (44). Knockdown of Endophilin B1 inhibited Bax/Bak conformational changes, cytochrome C release, cysteine asparaginase activation and cells death. This suggests that Endophilin B1 may represent a novel Bax activator that regulates mitochondrial apoptosis. In early stages of colorectal cancer patients, expression of Endophilin B1 was significantly lower than in controls. Inhibition of Bif-1 inhibits Bax/Bak activation, PI3KC3 activation and suppresses autophagy (67). Bif-1 haploinsufficiency attenuates mitochondrial autophagy, leads to up-regulation of Mcl-1, and inhibits Myc-induced caspase-3 activation in lymphoma cells (93). Above findings suggest the tumor suppressor function of Endophilin B1, and Endophilin B1 may be a promising target for treatment of cancers.

### Cardiovascular diseases

Early stages of atherosclerosis (AS) is mainly characterized by subendothelial deposition of oxidized low-density lipoprotein (oxLDL) in the vasculature, by which formation of macrophage-derived foam cells (MFCF) is a key in oxLDL deposition (94). OxLDL can trigger interaction of Endophilin A2 with CD36 or SR-A (scavenger receptor-A), and then induce activation of apoptosis signaling-regulated kinase-1 (ASK-1)/JNK/p38 signalings. Activated ASK-1/JNK/p38 signalings in turn up-regulated expression of CD36 or SR-A, promoting binding of oxLDL to the cell membrane and formation of MFCF (62). Thus, blocking Endophilin A2 may inhibit deposition of oxLDL and act as a potential treatment of AS.

Endophilin A2 is expressed in some eukaryotic cells, such as smooth muscle cells, tumor cells and neuronal cells. Endophilin A2 is able to function as a potential activator of autophagy. Endophilin A2 overexpression promotes formation of autophagosomes by binding Bif-1 to Beclin-1, which enhances autophagy and inhibits H_2_O_2_-induced apoptosis in H9C2 cardiomyocytes (41). In addition, Endophilin A2 protects against H_2_O_2_-induced apoptosis in basilar artery smooth muscle cells by regulating translocation of Bax from the cytoplasm to the mitochondria. Overexpression of Endophilin A2 attenuated cardiomyocyte apoptosis and reduced endoplasmic reticulum stress in response to myocardial infarction (MI) injury (95). In addition, Endophilin A2 knockdown led to increased vasodilation. When Endophilin A2 is silenced, addition of 17β-Estradiol (E2) increases expression of mERα. E2 forms a complex with mERα to activate the receptor tyrosine kinase, which then phosphorylates Endophilin A2 and attenuates its endocytosis. Finally, the process will stabilize the position of ERα in the lipid membrane (96). Thus, Endophilin A2 is associated with cardiovascular diseases progression.

### Autoimmune diseases

Dysfunction of antigen-presentation ability of B cells is associated with development and progression of autoimmune diseases. Antigen-specific B cell responses require endosomal transport to regulate antigen uptake and present antigen to helper T cells. The most characterized mechanism for internalization of B cell receptors (BCRs) from cell surface is Clathrin-mediated endocytosis (97). Activated B cells require iron uptake via endocytosis of transferrin receptors to maintain mitochondrial respiration (98). Endophilin A2-regulated intracellular transport is important for B cell-mediated humoral immunity by regulating antigen uptake, endocytosis homeostasis and cellular metabolism.

Rheumatoid arthritis (RA) is a systemic autoimmune disease characterized by synovial hyperplasia, development of vascular opacities, cartilage and bone degeneration (99). Mechanism of bone destruction in RA is related to proliferation and differentiation of osteoclasts. Bif-1 may modulate bone homeostasis by controlling the differentiation and function of osteoclasts. In the presence of nuclear factor‐κB (NF‐κB) receptor activator ligand (RANKL) and macrophage colony-stimulating factor (M-CSF), osteoclast production was accelerated in Bif-1 gene knockout mice, and the expression of osteoclast differentiation markers such as NFATc1, matrix metalloproteinase 9 (MMP-9), and cathepsin K (Cath K) was also increased. In contrast, overexpression of Bif-1 in RAW-D cells inhibits RANKL-induced osteoclast production, possibly due to disruption of the balance between pro-apoptotic and anti-apoptotic proteins. These results suggest that Bif-1 is a negative regulator of RANKL-induced osteoclast generation. It may be a promising target for the treatment of RA and osteoarthritis (100). There are excessive T cells in synovium of RA patients. Endophilin A2 triggers and drives autoimmune diseases such as RA by regulating T cell receptor internalization and activation of auto-reactive T cells. Collagen-induced arthritis (CIA) is a model of human RA with similar histopathological and clinical features. Endophilin A2 gene deficient mice reported reduced total joint scores, relieved inflammation and less development of arthritis. There was increased expression of Endophilin A2 in CD4^+^ T cells from CIA mice and RA patients compared to that in wild-type mice, healthy controls, suggesting that Endophilin A2 may regulate T cells activation. Similarly, deficiency of Endophilin A2 in experimental autoimmune encephalomyelitis (EAE) mice showed alleviated histopathology (63). Thus, Endophilin A2 is potential for autoimmune diseases targeting.

## Summary and outlook

Endophilin is involved in regulation of different biological functions, such as mitochondrial metabolism, apoptosis, and autophagy. Moreover, Endophilin performs significantly in the pathogenesis of neurodegenerative diseases, tumors, cardiovascular diseases, and autoimmune diseases. We have discussed the biological functions of the Endophilin family and how they lead to the occurrence and development of diseases. However, the specific mechanism of Endophilin involved in the pathogenesis of diseases is still unclear. There are still a number of issues that deserve attention. First, there is a lack of precise mechanism of Endophilin in endocytosis, such as how membrane curvature is generated? In addition, clear biological functions of Endophilin family in diseases progression are unknown. Thus, multicenter, large population-based studies are needed to explore expression profile of Endophilin in diseases, and more functional studies are warranted to clarify role of Endophilin in diseases pathogenesis.

## Author contributions

LY: Conceptualization, Formal analysis, Writing – original draft. AH: Conceptualization, Formal analysis, Writing – original draft. WX: Conceptualization, Data curation, Formal analysis, Writing – original draft.
